# Do Automatic Habits Predict Physical Activity Engagement in Older People?

**DOI:** 10.1093/geroni/igaa057.1602

**Published:** 2020-12-16

**Authors:** Sophie Andrews, Dinaz Parekh, Brooke Brady, Kim Delbaere, Simon Killcross, Kaarin Anstey

**Affiliations:** 1 Neuroscience Research Australia, Randwick, New South Wales, Australia; 2 Neuroscience Research Australia, Randwick, Australia; 3 University of New South Wales, Kensington, Australia; 4 University of New South Wales, Sydney, Australia

## Abstract

Sufficient physical activity is crucial to maintaining independence, health and wellbeing during ageing, yet physical inactivity is common in older people. Identifying factors associated with physical activity engagement is essential to develop novel approaches to increase activity in older people. Automatic, context-dependent habits may play an important role in physical activity behaviour. The current study aimed to investigate the relationship between physical activity behaviours and their automaticity in older people. 123 community dwelling Australians aged over 65 – 88 years (M=72.2; 81 women), recruited from participant registries, hospital noticeboards and community groups, completed an online questionnaire. Current physical activity levels were measured using the Incidental and Planned Exercise Questionnaire, and automaticity of those physical activity behaviours were measured using the Self-Report Habit Automaticity Index. Participants also reported demographic information, body mass index (BMI), medical history and current mood symptoms. Participants reported an average of 2.28 hours planned walking (SD=2.33), 5.81 hours planned moderate/vigorous exercise (SD=4.02), and 20.5 hours incidental activity (SD=15.52) per week. Multiple regression analyses revealed that after controlling for age, gender, BMI and depression symptoms, higher automaticity scores were associated with more hours per week of planned walking (p=.012), moderate/vigorous exercise (p=.038), and incidental activity (p=.017). Supporting older people to make their physical activity more habitual could therefore be an effective approach to increase levels of physical activity in this population.

